# Slowly developing depression of N-methyl-D-aspartate receptor mediated responses in young rat hippocampi

**DOI:** 10.1186/1471-2202-5-26

**Published:** 2004-08-03

**Authors:** Mikhail Dozmorov, Rui Li, Hui-Ping Xu, Barbro Jilderos, Holger Wigström

**Affiliations:** 1 Department of Medical Biophysics, Institute of Physiology and Pharmacology, Göteborg University, Box 433, SE 405 30 Göteborg, Sweden

## Abstract

**Background:**

Activation of N-methyl-D-aspartate (NMDA) type glutamate receptors is essential in triggering various forms of synaptic plasticity. A critical issue is to what extent such plasticity involves persistent changes of glutamate receptor subtypes and many prior studies have suggested a main role for alpha-amino-3-hydroxy-5-methyl-4-isoxazole propionic acid (AMPA) receptors in mediating the effect. Our previous work in hippocampal slices revealed that, under pharmacological unblocking of NMDA receptors, both AMPA and NMDA receptor mediated responses undergo a slowly developing depression. In the present study we have further adressed this phenomenon, focusing on the contribution via NMDA receptors. Pharmacologically isolated NMDA receptor mediated excitatory postsynaptic potentials (EPSPs) were recorded for two independent synaptic pathways in CA1 area using perfusion with low Mg^2+ ^(0.1 mM) to unblock NMDA receptors.

**Results:**

Following unblocking of NMDA receptors, there was a gradual decline of NMDA receptor mediated EPSPs for 2–3 hours towards a stable level of ca. 60–70 % of the maximal size. If such an experimental session was repeated twice in the same pathway with a period of NMDA receptor blockade in between, the depression attained in the first session was still evident in the second one and no further decay occurred. The persistency of the depression was also validated by comparison between pathways. It was found that the responses of a control pathway, unstimulated in the first session of receptor unblocking, behaved as novel responses when tested in association with the depressed pathway under the second session. In similar experiments, but with AP5 present during the first session, there was no subsequent difference between NMDA EPSPs.

**Conclusions:**

Our findings show that merely evoking NMDA receptor mediated responses results in a depression which is input specific, induced via NMDA receptor activation, and is maintained for several hours through periods of receptor blockade. The similarity to key features of long-term depression and long-term potentiation suggests a possible relation to these phenomena. Additionally, a short term potentiation and decay (<5 min) were observed during sudden start of NMDA receptor activation supporting the idea that NMDA receptor mediated responses are highly plastic.

## Background

Hippocampal synapses display a variety of activity dependent changes that may represent basic elements of memory. Of foremost interest are long-term potentiation (LTP) and depression (LTD), especially forms that depend on N-methyl-D-aspartate (NMDA) receptor activation and therefore can attain "associative" properties [1-3]. The selective induction of LTP versus LTD has been attributed to differing amounts of Ca^2+ ^ions entering via postsynaptic NMDA receptor channels [4]. Depending on type of stimulation, enzymes with different sensitivities to Ca^2+ ^may be engaged and change the balance between kinase and phosphatase activities, leading to either phosphorylation or dephosphorylation of postsynaptic target proteins, such as ionotropic receptors [2]. It has been shown that afferent stimulation by frequencies in the range 0.5 to 5 Hz reliably produces LTD whereas higher frequencies, 50–100 Hz, lead to LTP [5]. Several studies suggest that temporal factors are also important, implying that LTD requires a longer time to be induced than LTP [6]. We have previously demonstrated that under conditions of facilitated activation of NMDA receptors by low extracellular Mg^2+ ^synaptic plasticity can be induced by frequencies as low as 0.1–0.2 Hz when applied for prolonged periods of time [7]. Following an initial phase of transient potentiation there was a substantial depression that developed gradually during several hours and that remained stable after termination of NMDA receptor activation. Although the relation to "standard LTD" was not fully clarified, such slowly developing depression in low Mg^2+ ^solution may provide a useful model for studying certain forms of NMDA receptor dependent depression. In the present study, we will further develop the concept of gradually decaying responses.

One critical issue regarding LTP, LTD as well as other forms of glutamatergic synaptic plasticity, is the relative contribution of different glutamate receptor subtypes in creating the synaptic modification. Knowledge about this matter may be helpful in elucidating the underlying modification. While a selective change of alpha-amino-3-hydroxy-5-methyl-4-isoxazole propionic acid (AMPA) receptors has been cherished [8-10], especially in the case of LTP, several studies also observed NMDA receptor mediated changes in both LTP and LTD [11-14]. Previous work on LTD in our lab described an equal change of AMPA and NMDA responses [15]. However, it was reported by others that the relative contributions of AMPA and NMDA responses during LTD depend on experimental conditions, an equal change being one possible outcome [12]. In our recent examination of a slowly developing depression using composite AMPA-NMDA excitatory postsynaptic potentials (EPSPs) [7], the two responses declined in close parallel, indicating a common factor. Such an equal change is compatible with both a coordinated change of receptors and a presynaptic one via a decrease of glutamate release. However, in view of other studies reporting a coupling between responses via AMPA and NMDA receptors [16,17], one may ask whether our observation of declining NMDA responses could be secondary to the change of AMPA. In the present study, isolated NMDA receptor mediated EPSPs were shown to decline progressively during prolonged low frequency activation (0.1 Hz). Moreover, following sudden start of stimulation there was an initial, transient potentiation. Our findings also resolved some questions regarding input specificity and durability of the slow decay, which were previously addressed only for AMPA EPSPs.

## Results

### Isolated NMDA EPSPs show a progressive decay

**Figure 1:**
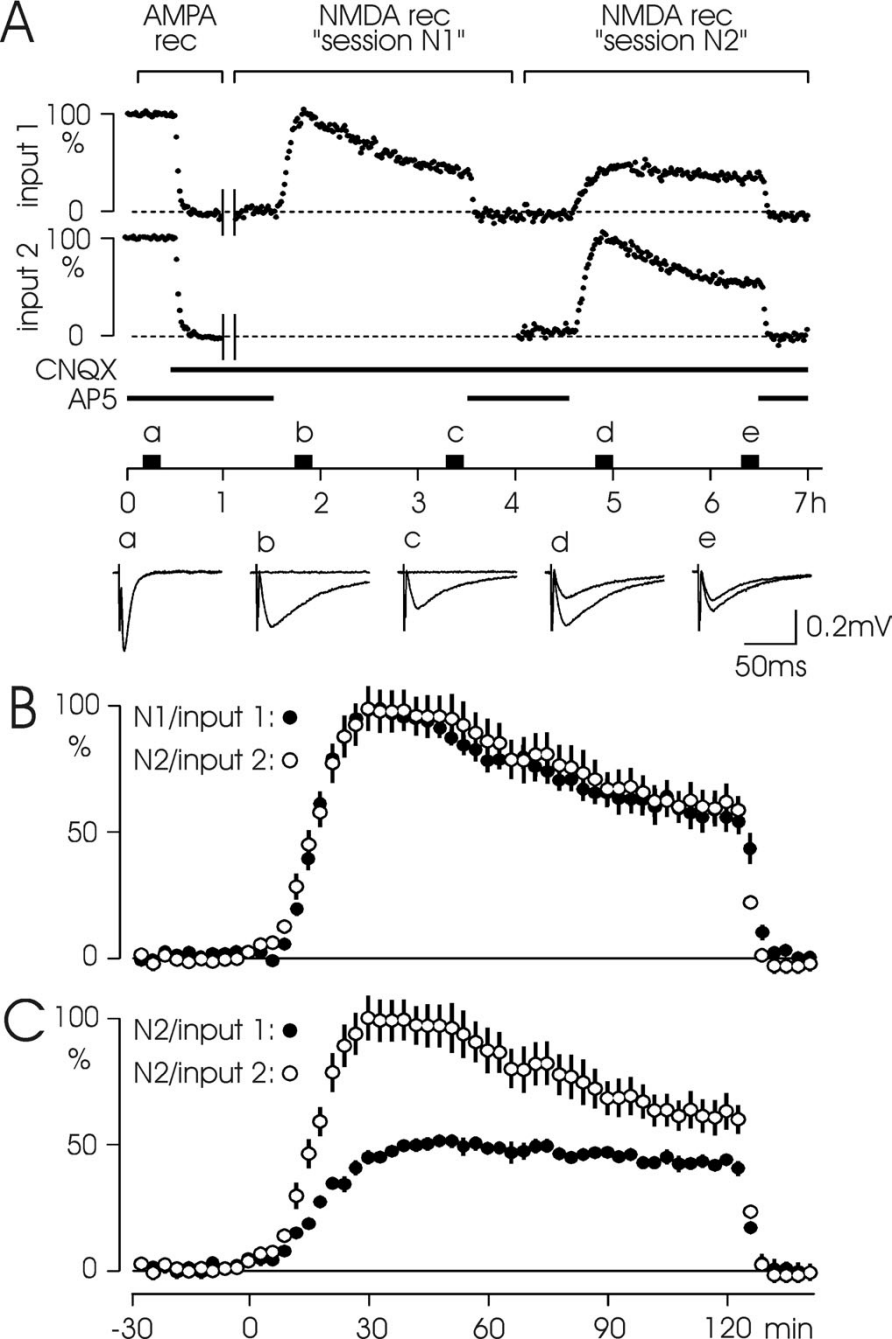
**Decay of NMDA receptor mediated EPSPs in low Mg^2+ ^solution. **(A) Experimental design: Measurements of field EPSPs from a representative experiment are plotted for two independent pathways, referred to as input 1 and input 2. By appropriate use of specific blockers CNQX and AP5, either isolated AMPA EPSPs (used for initial pathway equalization) or isolated NMDA EPSPs (used in testing sessions N1 and N2) were recorded with periods of fully blocked responses in between. The pathways were stimulated alternately, each at 0.1 Hz, except for silencing input 2 for a 3 h period that contained session N1. Each point represents the average of measurements within 1 min. As seen, during N1 the responses to input 1 decayed. During session N2 both inputs were stimulated, revealing a novel decay for input 2 and occluded depression for input 1. Samples of recorded potentials of both inputs are given for the indicated time points a-e. (B) Superimposed, averaged time courses of NMDA EPSPs for input 1 during session N1, and input 2 during session N2. (C) Superimposed averaged time courses of NMDA EPSPs for input 1 and input 2, both during session N2. For B and C, each point represents the average of measurements within 3 min intervals. The peak during session N1 was used as 100 %. Values are expressed as mean ± S.E.M (n = 8 experiments).

AMPA EPSPs were initially recorded in low Mg^2+ ^solution in the presence of NMDA receptor antagonist AP5 to allow for pathway equalization (see Methods) without evoking NMDA EPSPs. Synaptic transmission was then entirely blocked by adding AMPA receptor antagonist CNQX, followed by unblocking of NMDA receptors by wash out of AP5. During this time, only one pathway was stimulated, keeping the other one silent for later use. As illustrated in Fig. 1A (upper part), an NMDA receptor mediated EPSP appeared within 10 min and reached maximum about 30 min after switching to AP5-free solution. During the following recording period of nearly 2 h, the NMDA EPSP decayed substantially, on average down to 58 ± 6 % of peak value (n = 8). Control experiments showed that isolated AMPA EPSPs recorded in low Mg^2+ ^remained stable for several hours (changing to 96 ± 5 % of baseline after 2 h, n = 5, not illustrated).

### Reinduction in a naive pathway

To exclude the possibility that the observed decay of NMDA responses were due to deterioration of slices, implying a general decrease of essential physiological processes, the experiment was repeated for the other pathway, i.e. the one that had not previously expressed NMDA responses (continuation in the same set of 8 slices). As seen in Fig. 1A (lower part), a similar result was obtained as above. The NMDA EPSP peaked at 98 ± 7 % and declined to 63 ± 8 % relative to the peak in the first experimental session. As illustrated in Fig. 1B, the curve obtained for a naive pathway during the second session of NMDA unblocking was similar to the one obtained for the pathway activated during the first session, the two curves overlapping closely for the entire recording period.

### Comparison between pathways

#### Specificity and persistency

The same experiment was used to address the question of persistency as well as input specificity of the slowly developing depression of NMDA EPSPs. As can be seen in Fig. 1A, the pathway that was active during the first experimental session was retested during the second one together with the pathway receiving novel NMDA receptor activation. This allowed for a comparison between the pathways (for convenience, the peak in the first session is still used as reference for the values in the following). During the second session of NMDA receptor unblocking, the previously treated pathway displayed a substantially smaller peak than the naive one (48 ± 3 % vs. 98 ± 7 % p < 0.05, n = 8), and the two curves were still different by the end of the session (44 ± 3 % vs. 63 ± 8 %; difference 19 ± 4 %, p < 0.05). Since the latter time point was located at 3 h after the end of the first session it is evident that the depression of NMDA EPSPs lasted for at least 3 h. It is noteworthy that the previously depressed pathway showed no significant decay during the second session, passing from 48 to 44 %, i.e. a relative change by 92 % (p > 0.05), as if it had already been saturated. For a comparison, the naive pathway changed by a factor 63/98 = 64 % (p < 0.05) (see curves in Fig. 1C). A graphic summary of all "peak" and "end-of-session" values is given in Fig. 3B. It can also be noted that NMDA EPSPs recorded for contiguous time intervals up to 4 h reached a saturation level after 2–3 h (n = 3, not illustrated).

#### Instantaneous versus persistent depression

The above results suggest that the progressive decay observed in a single pathway as an instant event actually represents a long-term, pathway-specific change that can be assessed long later by comparison across pathways. To further examine the relation between the instantaneously recorded depression and the one measured about 90 min later, the relation between the two was plotted as illustrated in Fig. 3C. The two variables were found to be positively correlated (r = 0.71, p < 0.05, n = 8), implying that depression to a lower level in one pathway led to smaller responses in that pathway at later times compared to another pathway. The regression line, passing below rather than through the point of no depression (100 %, 100 %), indicates that even a slight instantaneous decay may be coupled to a noticeable change in the long term. Possibly, the declining trend was partially masked by recovery from AP5 leading to an underestimation of it.

### Effect of NMDA receptor blockade on subsequent NMDA EPSP decay

**Figure 2:**
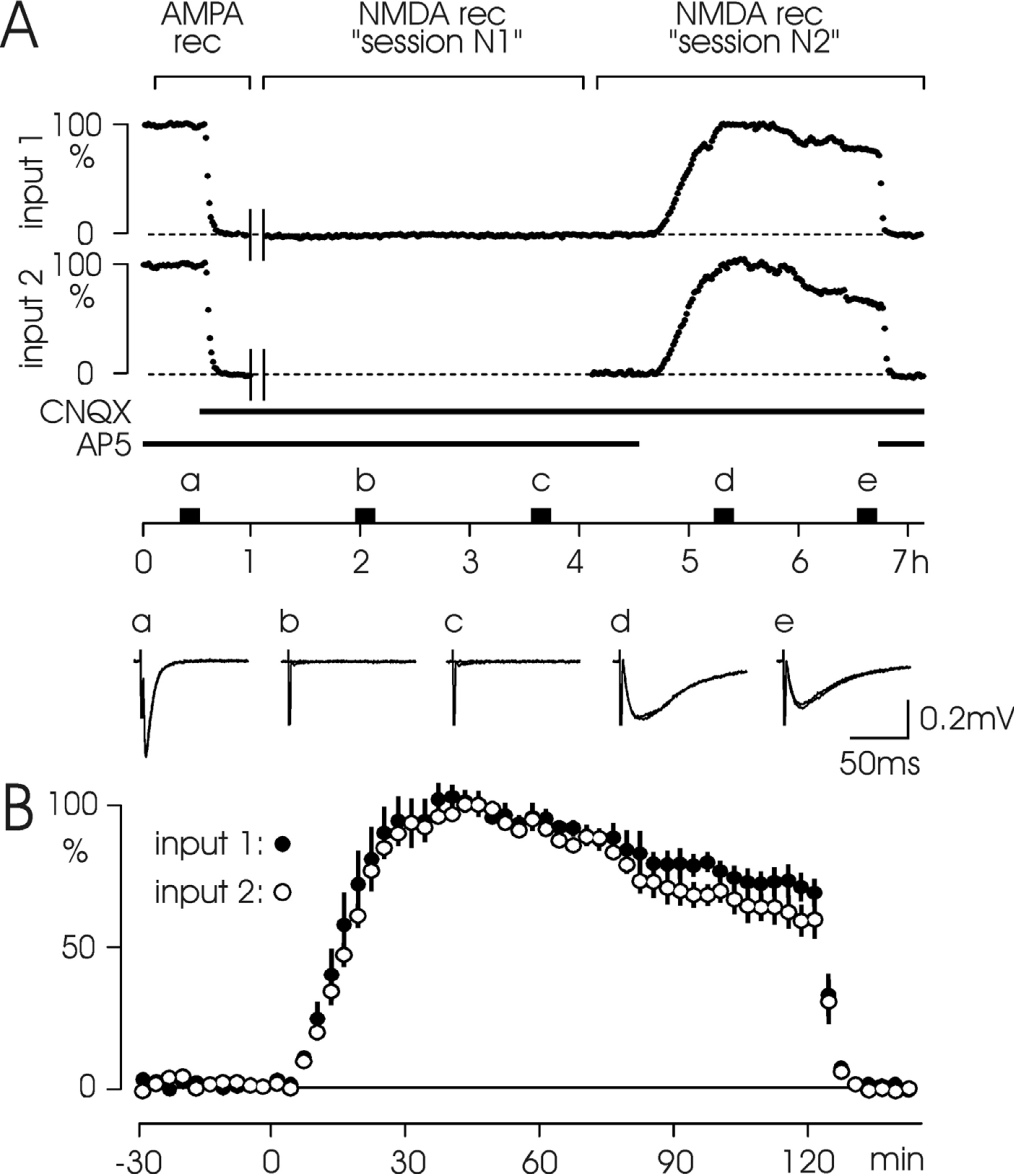
**Activation of NMDA receptors is necessary for inducing persistent depression. **(A) Experimental design: Measurements of field EPSPs are plotted for a representative experiment as in Fig. 1A. The experiment conformed with the previous one except that AP5 was present in the solution during session N1. Accordingly, synaptic transmission was blocked for the entire period when input 2 was stopped. In session N2, AP5 was eventually washed out and both inputs displayed decaying NMDA EPSPs. Samples of recorded potentials of both inputs are given for the indicated time points a-e. (B) Superimposed averaged time courses of NMDA EPSPs from both inputs during session N2. Each point represents the average of measurements within 3 min intervals. The peak of input 2 was chosen as 100 %. The equal appearance of the curves shows that no pathway specific depression remained during N2 due to the differential treatment during N1 (compare such a depression in the reference case in Fig. 1C). Values are expressed as mean ± S.E.M (n = 5 experiments).

**Figure 3:**
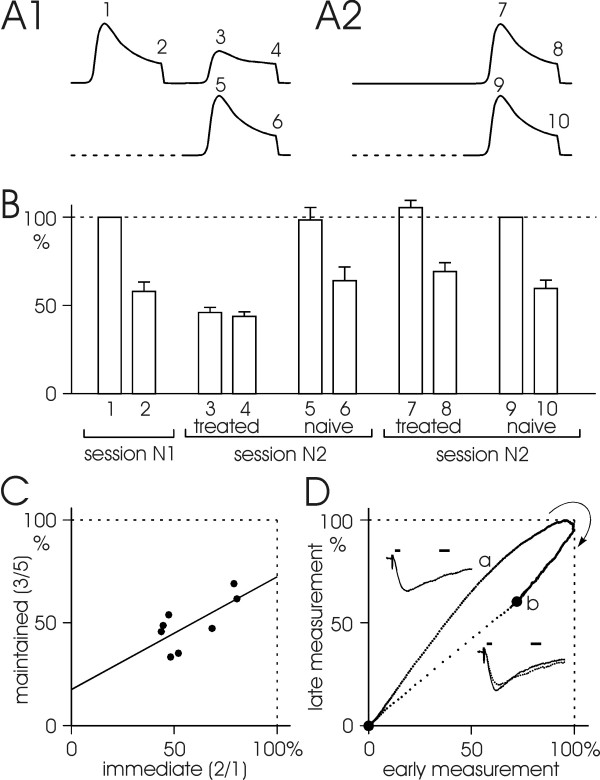
**Summary of NMDA EPSP depressions during unblocking of NMDA receptors. **(A1) Schematic representation of the experiment described in Fig. 1. (A2) Schematic representation of the experiment described in Fig. 2. Pathways are defined as "treated" or "naive" depending on whether stimulation was "on" (solid) or "off" (dashed) during session N1. Each number (1–10) in A1 and A2 represents a portion where measurements were taken for analysis. (B) Comparison between NMDA EPSP measurements, each bar representing an average obtained for 30 consecutive responses (5 min). Value no. 1 was chosen as 100 % for experiments depicted in A1. Since a corresponding value was lacking for experiments depicted in A2, value no. 9 was chosen as 100 %. All values are expressed as mean and S.E.M (n = 8 and n = 5, respectively, for A1 and A2 type experiments). (C) Relation between instantaneous depression in session N1 (abscissa, final value relative to peak) and persistent depression in session N2 (ordinate, interpathway comparison of peaks). Each dot represents a single experiment. The analysis revealed a significant correlation between the variables as illustrated by the superimposed regression line (n = 8). (D) Relation between early and late measurements obtained with two different time windows (initial 5 ms and 35–45 ms, respectively) plotted as a "phase trajectory" (abscissa, early measure; ordinate, late measure). The curve represents an average of 20 experiments with additional smoothing to reduce noise (5 min moving average). Samples of recorded NMDA EPSPs corresponding to locations a, b of the trajectory are overlaid. Bars above EPSPs curves indicate time windows used for early and late measurements, respectively. The curve at b is shown superimposed on a dotted copy of the curve at a.

To pinpoint the induction mechanism, in terms of a pre- versus postsynaptic location, experiments were carried out in a similar way as above except for keeping AP5 in the solution during the first session (see Fig. 2A). Hence, the stimulus pattern included a 3 h long interval with no stimulation in one of the pathways. The other pathway was stimulated during that time, and most likely releasing glutamate, but no postsynaptic response was expressed due to blockade of NMDA receptors. It can be argued that successful blockade of depression would predict a postsynaptic mechanism whereas a failure to do so would predict a presynaptic one. Fig. 2B shows that the depression was actually blocked, demonstrating the importance of NMDA receptor activation in the induction process. As illustrated, the two curves obtained during unblocking of NMDA receptors in the second session were quite similar. The continuously stimulated pathway, being depressed in the standard case, peaked at a level of 105 ± 5 % relative to the control pathway (n = 5; see also graphic summary of values in Fig. 3B).

### Phase trajectories as indicators of waveform change

Depending on the type of synaptic modification, EPSP waveforms may change in different ways, and previous work in our lab has demonstrated that NMDA EPSPs are more prone than AMPA EPSPs to show these changes [18]. Taking advantage of such waveform analysis, which might shed light on the underlying mechanism, we examined phase plots based on measures of the initial part and the later part of the NMDA EPSP (see Methods) on the X- and Y-axis, respectively. The curve in Fig. 3D is based on a total of 20 experiments (pathways), including some with only a single session. As shown, the phase trajectory displayed a loop indicating a difference between the effect of AP5 and the gradual decay of NMDA EPSPs. On the average the encircled area was 15 ± 2 % (p < 0.05, n = 20; scaling peak × peak as 100 %, clockwise being positive).

While our data show that the time window matters for measuring NMDA EPSPs, all the above results were qualitatively similar regardless of which window was used. It can be noted, however, that the depression of NMDA EPSPs by the end of a recording session was less pronounced using early measurements than late ones (responses attaining 72 ± 3 % vs. 61 ± 3 % of the peak value, p < 0.05, n = 20).

In 12 out of the 20 experiments, the fiber volley was well separated from the stimulus artifact allowing it to be properly measured. No significant change was detected, the end-of session value amounting 103 ± 2 % of the value at the EPSP peak (p > 0.05, not illustrated).

### Short-term effects induced by sudden onset stimulation

**Figure 4:**
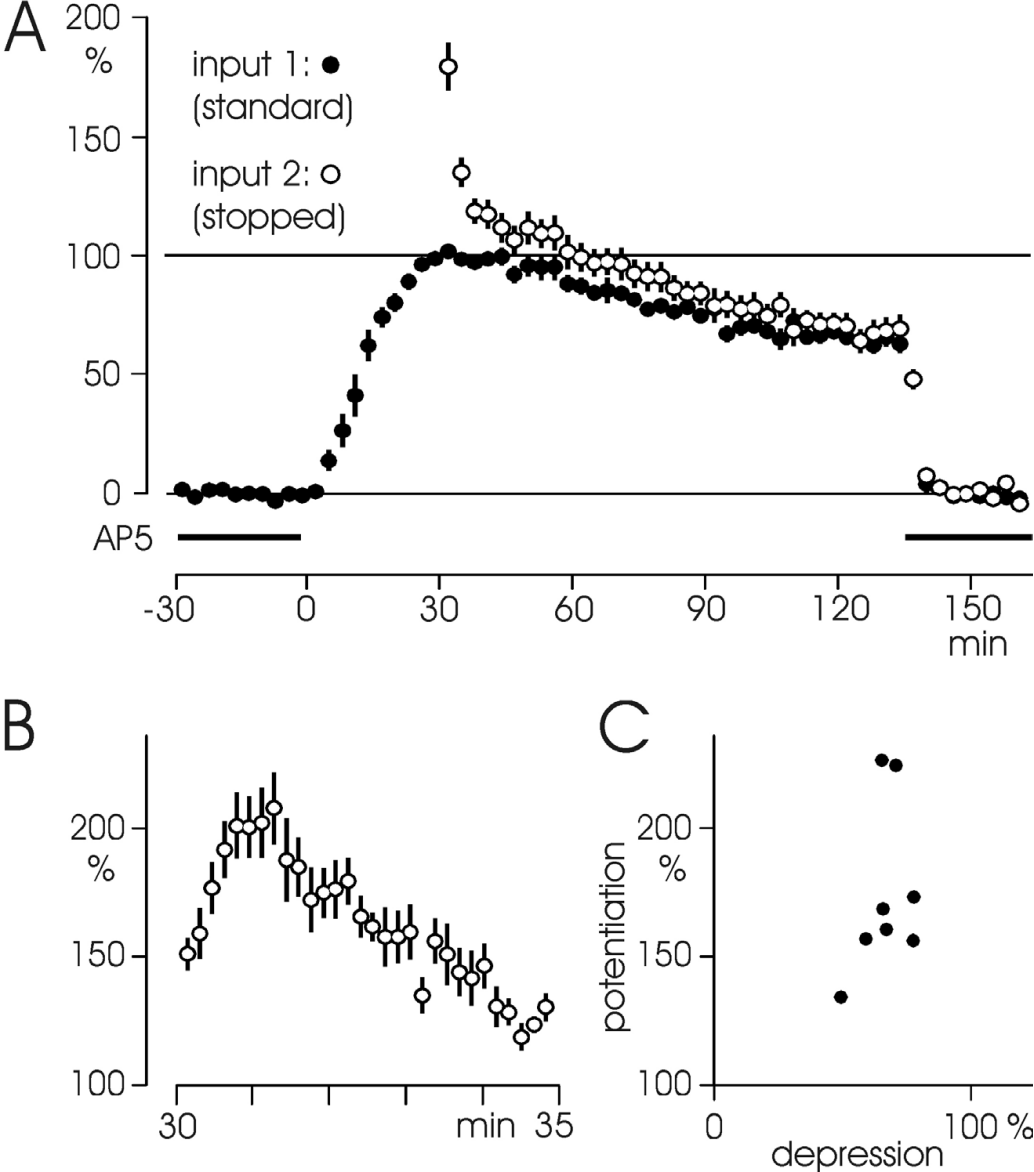
**Transient potentiation induced by sudden activation of NMDA receptors. **(A) Superimposed averaged time courses of NMDA EPSPs for two pathways subjected to different stimulation protocols. Each point represents the average of measurements within 3 min intervals. Stimulation of one pathway (input 2) was stopped for a period of 30 min and then restarted at a time point when NMDA receptors were fully unblocked as judged by responses for the other pathway (input 1) that was exposed to the standard protocol. The peak of input 1 was taken as 100 %. (B) Time course of the newly evoked NMDA responses. Expanded portion of A immediately following restart of stimulation, each point representing measurement of a single EPSP. It can be noted that following the initial response there was additional growth for about a minute before responses started to decay. Values in both A and B are expressed as mean ± S.E.M (n = 8 experiments). (C) Examining the relation between the slow depression and the transient potentiation of NMDA EPSPs. Depression was quantified as end-of-session value relative to peak value (5 min averages) for input 1 that received the standard protocol. Transient potentiation was estimated as the average of the first 18 responses (3 min) of input 2 relative to the average of the corresponding interleaved responses of input 1. The latter value was nearly the same as the peak value. Each dot represents a single experiment (same set of 8 experiments as in A, B). The analysis revealed no significant correlation between the data points.

In the above, NMDA receptor activation occurred gradually while the antagonist AP5 was washed out. This is in line with the experimental protocol used in our previous work on composite EPSPs containing both AMPA and NMDA components [7]. However, a natural question is whether sudden, novel activation of NMDA receptors is equivalent in producing the results observed here. We therefore pursued experiments in which stimulation was silenced until washout of AP5 was complete. One pathway, receiving such sudden stimulation, was compared to a control pathway subjected to gradual NMDA receptor activation during a single recording session. Fig. 4A shows an essential difference in behavior between the pathways, the sudden start of activation leading to substantially larger responses for about 5 min. Fig. 4B reveals additional complexity, the initial responses showing actual growth of responses for about a minute before they started to decay, implying an early potentiation process. The total range of responses was substantial, from a peak above 200 % to about 70 % by the end of the recording session (relative to the peak of the control pathway), i.e. about 3 times.

In order to determine whether the transient potentiation had any obvious relation to the slow depression of NMDA EPSPs, the relation between the two was examined. Thus, the degree of initial potentiation was calculated by comparing the pathways just after stimulation was started and the depression was determined, as before, by comparing the end-of-session value with the peak value (see legend of Fig. 4 for further details). The two variables, illustrated by the XY-plot in Fig. 4C, were found to have no significant correlation (r = 0.35, p > 0.05).

## Discussion

Our study revealed a progressive decline of pharmacologically isolated NMDA EPSPs, as observed for several hours in response to low rate (0.1 Hz) activation of afferents. The decline was found to be a form of long-term synaptic depression with an induction linked to NMDA receptor activation and with an expression that was maintained through periods without such activation. Several of its basic characteristics were similar to those of conventional LTP and LTD, suggesting a possible relation to these phenomena.

### Synapse specificity and NMDA-dependent induction

Decaying responses is a potential side effect in long-term, electric recording *in vitro *due to declining viability of biological tissue or other experimental imperfections. Such unspecific "run down" can not account for the present findings since the gradual depression of responses could be repeated in the same slice, using a previously undepressed pathway. On the other hand, if the experiment was repeated twice in the same pathway, the second occasion revealed a diminished NMDA EPSP that showed little further decay. Together, these results show that the depression is input specific and long lasting and that it can saturate. Moreover, the lack of associated changes of the fiber volley speaks against a failure of axon conductance [19], favoring a synaptic localization of the process.

While both pre- and postsynaptic expression mechanisms appear feasible, certain mechanisms of induction can be excluded. For instance, a decrease in probability of glutamate release due to a direct depletion of the vesicle pool is unlikely since AMPA EPSPs could be evoked for several hours without significant decay (see also [7,20]). Even so, a use-dependent reduction of vesicle content may affect NMDA responses selectively under certain conditions by restricting "glutamate spillover" [21]. The most critical data with respect to the induction mechanism is that a period of conditioning stimulation, normally leading to reduction of NMDA EPSPs in the same pathway later on, was ineffective if delivered during blockade of NMDA receptors. This implies that the induction of the depression requires activation of NMDA receptors, most likely postsynaptically.

### Other observations of decaying NMDA EPSPs

The input specificity and NMDA dependent induction of the current depression conform with basic properties of conventional LTP and LTD [22]. The depression might then be a case of LTD, although induced by an alternative protocol. In fact, LTD was shown to be associated with changes involving both AMPA and NMDA receptors, although the linkage between the two contributions is controversial [12,15]. Moreover, both of the cited studies demonstrated LTD of isolated NMDA EPSPs induced by 1–2 Hz stimulation. In contrast, experiments in cultures, inducing LTD by field stimulation at a higher frequency (5 Hz), reported only AMPA receptor mediated changes [10]. Direct interaction tests may further clarify the relation between the present depression and LTD.

Gradually decaying, NMDA receptor mediated responses were observed previously in our lab during recording of composite AMPA-NMDA EPSPs for several hours [7,23]. Attempts to relate the decay to LTD demonstrated a weak reduction of subsequent LTD of AMPA responses suggesting at least some elements in common [7]. In view of studies reporting forms of AMPA-NMDA coupling [16,17], it is arguable that the studies demonstrating a decay of both components could have been influenced by the use of composite responses. In one of our studies [7], the observed depression of the AMPA component of composite EPSPs was verified by additional comparison between isolated AMPA EPSPs obtained under blockade of NMDA receptors. A similar verification was lacking for the depression of the NMDA response. By recording of isolated NMDA EPSPs, the present study ascertains that NMDA receptor mediated responses undergo a use-dependent depression, which is manifested in the absence of AMPA receptor activation. However, the decay was less pronounced than that reported previously for the NMDA component of composite EPSPs (average reduction to 60 % of peak as compared to 40 % in the previous study [7]).

While we observed that isolated NMDA EPSPs decay "spontaneously", most prior studies employing such EPSPs did not report a decay. It might be that limitations of recording time concealed the effect and cell dialysis during whole cell recording could also be a limiting factor. Actually, a recent study, recording "novel" responses under whole cell conditions, reported on decaying AMPA EPSPs but constant NMDA EPSPs [24]. The possibility of AMPA receptor LTD under the present conditions could not be excluded as the blockade of the receptors may just conceal the effect. Further studies may help to reveal this matter.

### Persistency and saturability

Standard LTP/LTD experiments compare relatively stable periods of recording before and after induction of the synaptic modification. This was not possible in the present case, since merely test stimulation evoked the decay. Therefore, comparisons were generally made between synaptic pathways subjected to different stimulus paradigms. The induction of depression in a single pathway during an initial 2 h period caused a subsequent difference between NMDA responses of the two pathways throughout a subsequent test period. The degree of initial decay was closely related to the later difference between pathways, suggesting that once depression occurred it could be maintained through periods of receptor blockade until testing was performed. Our data suggest a duration of the depression of more than 3 h after the initial induction period. This is in the range commonly referred to as "late", which is believed to involve special biochemistry such as gene expression and protein synthesis [25,26]. Whether, the presently studied depression involves such changes remains to be determined.

The gradual depression of NMDA EPSPs was found to saturate after 2–3 h as evidenced by both single and double session experiments. This is in line with several other forms of NMDA-dependent plasticity, including LTP, LTD and chemically induced variants, which are shown to be saturable [27-29]. Whether, the saturation observed here is a "true one" at the level of expression is not known. Alternatively it could be a phenomenon at the induction level, related to weaker induction due to the diminished NMDA response.

### Possible expression mechanisms

Previous work on conventionally induced LTD revealed an essential role for protein phosphatases in mediating the synaptic modification [30,31]. Consistent with the idea that changes of AMPA receptors mediate NMDA-dependent synaptic plasticity [32,33] it was demonstrated that certain sites of the GluR1 subunit were targeted in LTP/de-potentiation and other ones in LTD/de-depression [2,34]. Less is known about mechanisms underlying NMDA receptor changes in LTP/LTD as well as in the current depression. A previous study in our lab recording composite EPSPs reported that LTD of the NMDA component was blocked by a phosphatase inhibitor in a similar manner as "standard LTD" [15]. Hence, one can envisage that NMDA receptors would be controlled via dephosphorylation in a similar manner as inferred for AMPA receptors.

NMDA receptors also have a number of other regulatory sites, allowing for modulation by glycine, polyamines, calcium, and redox agents [35] and they have shown to be mobile as well [36-38], in keeping with the idea of mobile AMPA receptors in LTP/LTD [39,40]. Regardless of details, additional factors are needed to stabilize the synaptic modification in the long term, perhaps via synthesis of new proteins as previously demonstrated for LTP and LTD lasting longer than about 3 h [25,28,41]. Changes in synaptic morphology and altered subunit composition of receptors are examples of protein synthesis dependent mechanisms that have been implied in late forms of plasticity [32,42].

Although a postsynaptic modification appears to be the primary choice, a presynaptic one that is initiated postsynaptically is also conceivable. In previous attempts to distinguish between pre- and postsynaptic mechanisms, LTD was compared with depression caused by various pharmacological agents with respect to the ability to influence the waveform of EPSPs [18]. While LTD in that study was found to affect isolated NMDA EPSPs in a uniform manner, i.e. no waveform change, the present data appeared to be less clear-cut. Nevertheless, the relation between early and late EPSP measurements differed for the initial AP5 washout period and the following period of actively induced depression, indicating a change in EPSP waveform. The depression therefore appeared to be distinct from a postsynaptic modification via modulation of channel gating. However, a clear test of the pre-post issue still remains. Unfortunately, the MK-801 test of release probability [43] does not appear useful when dealing with decaying responses as in the present case.

### Short-term changes and their possible mechanisms

While the main line of experiments employed a smooth start of NMDA receptor activation following the gradual washout of AP5, another set of experiments made use of sudden activation by awaiting full washout until stimulation was started. Compared to smooth activation, there was an additional, transient potentiation that largely decayed within 20–30 stimuli. This is in accord with a previous study in hippocampal slices showing that stopping stimulation of composite AMPA-NMDA EPSPs for 10–60 min (and one case of isolated NMDA EPSP for 10 min) resulted in a transient potentiation when stimulation was resumed [23].

Several other studies describe decaying NMDA responses in relation to inactivation or desensitization of receptors [44,45]. Accordingly, synaptically evoked NMDA responses in cell cultures were found to inactivate (i.e. decay) within a few minutes in much the same manner as observed here [45], a process shown to be triggered by postsynaptic influx of Ca^2+ ^via the NMDA channels. Similar mechanisms of receptor desensitization/inactivation might be responsible in the present case in forming the transient phase after starting stimulation. Some details remain unexplained by this simple model, such as the biphasic character of the transient phase in terms of initial growth and subsequent decay. One can speculate that a minor LTP, or short-term potentiation, might be induced by the sudden activation of NMDA receptors and so would contribute to the initial growth, although the underlying cause is not addressed in this kind of explanation.

## Conclusions

The above results emphasize that NMDA receptor mediated responses are highly plastic and that mere test stimulation can induce a short-term potentiation as well as a slowly developing depression that persists for several hours. The depression was input specific and saturable, and its induction required NMDA but not AMPA receptor activation in conformity with conventionally induced LTP and LTD, suggesting a relation to these phenomena. While a low Mg^2+ ^solution was used in our case to unblock NMDA receptors, similar unblocking may occur naturally in response to depolarization. Several important issues are still not settled. Is the saturation of the NMDA EPSP depression an absolute matter or can it be overcome, leading to further down regulation and possibly silencing of synapses? Conversely, is it possible to reverse, i.e. de-depress, the change by LTP or similar processes, allowing for bidirectional control? Further research is needed to resolve these questions.

## Methods

Experiments were performed on 12 to 18 day old Sprague-Dawley rats. The animals were decapitated after isoflurane (Forene) anesthesia in accordance with the guidelines of the Swedish Council for Laboratory Animals. All animal procedures were approved by the Local Ethics Committee at Göteborg University. The brain was removed and placed in an ice-cold artificial cerebrospinal fluid solution containing (in mM) NaCl 119, KCl 2.5, CaCl_2 _2, MgCl_2 _2, NaHCO_3 _26, NaH_2_PO_4 _1 and glucose 10, oxygenated by 95% O_2_, 5% CO_2_. The hippocampus was dissected out and transverse 400 μm thick slices were prepared by a vibratome or tissue chopper. The slices were initially kept in the same solution at room temperature for at least 60 min. As required, slices were then transferred to one or several "submerged type" recording chambers. During the experiment, slices were perfused at 30°C by a solution similar to that above except that the concentration of Mg^2+ ^was 0.1 mM. The usage of low Mg^2+ ^allowed for expression of NMDA receptor mediated responses.

Stimulation was delivered as 0.1 ms negative constant current pulses via monopolar tungsten electrodes. For each slice, two stimulating electrodes were placed in the apical dendritic layer of CA1 pyramidal cells on either side of the recording electrode to provide for stimulation of two separate sets of afferents. Field EPSPs were recorded by using a glass micropipette filled with 3 M NaCl (4–10 MΩ resistance). The basal test stimulus frequency was 0.1 Hz with stimuli delivered alternately to the two electrodes, successive stimuli being separated by 5 s. To test the effect of stimulus interruption, one of the two electrodes was given no stimulation during a certain time, the other one remaining stimulated at 0.1 Hz.

Recording commenced by monitoring isolated AMPA EPSPs in the presence of AP5 (50 μM) to block NMDA responses. A low concentration of CNQX (1 μM) was used to partially suppress the AMPA responses. In this way, somewhat larger stimulus strengths could be applied, suitable for evoking isolated NMDA EPSPs in the later part of the experiment. During the time of AMPA EPSP recording, the stimulus strengths were adjusted for each slice to equalize the synaptic inputs of the two pathways. This was essential for later comparison of NMDA EPSP across pathways. After obtaining a baseline of equal AMPA responses, the concentration of CNQX was raised to 10 μM which entirely blocked synaptic responces. The remaining non-synaptic response, consisting of stimulus artifact and presynaptic volley, was used to define "true zero".

To study NMDA receptor mediated responses, CNQX (10 μM) was maintained in the solution while AP5 was washed out for one or several 2 h periods, referred to as sessions in the following. In between the sessions as well as afterwards, synaptic transmission was again blocked by applying AP5 (50 μM), framing in the sessions by periods of recording non-synaptic responses. Under the sessions, various tests were made depending on the purpose of investigation. Usually one input remained silent during the first session and stimulation was not resumed until after synaptic transmission was reblocked. In another kind of experiment, the initially silent pathway was reactivated in the early part of the first session after NMDA receptors were unblocked, providing a means for sudden start of NMDA receptor activation.

Signals were amplified, filtered and transferred to a PC clone computer for on-line and off-line analysis by specially designed electronic equipment (based on an Eagle Instruments multifunction board) and own developed computer software. AMPA EPSPs were measured using an early time window (first 1.5 ms after the fiber volley) while NMDA EPSPs were measured using both an early (first 5 ms after volley) and a late (35–45 ms after artifact) time window. The late measurement was used in presenting most of the results, allowing easy comparison with previous work in our lab that estimated the NMDA component of composite EPSPs via a late measurement [7]. Similar albeit not identical results were obtained with early and late measurements (see illustration in Fig. 3D).

Measurements were calculated by integrating the curve along the specified time window after substraction of the prestimulus baseline. All values were corrected by substracting the corresponding measurements of the non-synaptic potential obtained after total blockage of the EPSPs (except when measuring the fiber volley). The final data were quantified as relative values compared to a reference level defining 100 %. While the initial baseline formed a natural reference for AMPA responses, the choice was less obvious for NMDA responses, leading us to use the highest level of responses for one of the pathways in one of the experimental sessions (selected to make sense). Results are expressed as mean ± S.E.M. Statistical comparisons were made using Student's t-test.

Drugs were obtained from Tocris Cookson, UK; prefabricated stimulating electrodes were obtained from World Precision Instruments, FL USA, type TM33B.

## Abbreviations

AMPA, alpha-amino-3-hydroxy-5-methyl-4-isoxazole propionic acid;

AP5, D(-)-2-amino-5-phosphonopentanoic acid;

CA, cornu ammonis;

CNQX, 6-cyano-7-nitroquinoxaline-2,3-dione;

EPSP, excitatory postsynaptic potential;

LTD, long-term depression;

LTP, long-term potentiation;

NMDA, N-methyl-D-aspartate;

## Authors' contributions

MD planned and carried out most of the experiments including data analysis, and compiled the manuscript. RL carried out experiments, participated in the planning process and helped in shaping the final manuscript. HPX carried out the initial experiments establishing the effect of NMDA EPSP depression. BJ was responsible for logistics planning and participated in experiments. HW conceived of the study, and participated in its design and coordination. All authors read and approved the final manuscript.
